# CHANGES IN THE PSYCHOLOGICAL COSTS OF MATERNAL FAVORITISM FOR DAUGHTERS AND SONS AS MOTHERS AGE

**DOI:** 10.1093/geroni/igz038.1530

**Published:** 2019-11-08

**Authors:** J Jill Suitor, Megan Gilligan, Marissa Rurka, Yifei Hou, Catherine Stepniak

**Affiliations:** 1 Purdue University, West Lafayette, Indiana, United States; 2 HDFS, Iowa State University, Ames, Iowa, United States

## Abstract

Life course perspectives suggest that the consequences of being mothers’ favorite children will vary, depending on the expectations associated with that status at different points in mothers’ lives. We propose that maternal favoritism predicts depressive symptoms only when mothers are older and at greater risk of facing losses for which favored children perceive they should provide additional emotional support. To address this question we used mixed-methods panel data collected from 479 adult children as part of the Within-Family Differences Study. Multi-level regression analyses revealed that perceiving oneself as the child most emotionally close to the mother did not predict depressive symptoms for daughters or sons at T1, but was a predictor of daughters’ depressive symptoms at T2. Qualitative analyses revealed that by T2, favored daughters had begun perceiving themselves as emotional caregivers when mothers faced age-related losses, whereas favored sons did not hold these role perceptions at either wave.

